# Factors Influencing the Formation of Chemical–Hemoglobin Adducts

**DOI:** 10.3390/toxics10010002

**Published:** 2021-12-21

**Authors:** Yuko Shimamura, Akina Okuda, Kenya Ichikawa, Ryo Inagaki, Sohei Ito, Hiroshi Honda, Shuichi Masuda

**Affiliations:** 1 School of Food and Nutritional Sciences, University of Shizuoka, 52-1 Yada, Suruga-ku, Shizuoka 422-8526, Japan; shimamura@u-shizuoka-ken.ac.jp (Y.S.); s19203s@u-shizuoka-ken.ac.jp (A.O.); 18205@u-shizuoka-ken.ac.jp (K.I.); s16601@u-shizuoka-ken.ac.jp (R.I.); itosohei@u-shizuoka-ken.ac.jp (S.I.); 2 R&D Safety Science Research, KAO Corporation, 2606 Akabane, Ichikai-Machi, Haga-Gun, Tochigi 321-3497, Japan; honda.hiroshi@kao.com

**Keywords:** hemoglobin (Hb) adducts, glycidol, acrylamide, glucose

## Abstract

Hemoglobin (Hb) adducts have been used as biomarkers for the internal exposure to chemicals. Simultaneous exposure to chemicals that bond with the N-terminal valine of Hb to form adducts, such as glycidol, acrylamide, and glucose, may affect the formation of the individual Hb adducts. In this study, various factors influencing the formation of chemical–Hb adducts were analyzed using in vitro and in vivo systems. In the in vitro assays, the formation of glycidol– and acrylamide–Hb adducts was altered in the presence of glucose, serum albumin, and other chemicals. In contrast, in the in vivo experiments, glycidol– and acrylamide–Hb adduct formation was unchanged in mice exposed to glycidol and acrylamide. The interaction between glycidol and acrylamide with residues other than the N-terminal valine of Hb was analyzed using the protein thermal shift assay. Glycidol and acrylamide also interacted with amino acid residues other than the N-terminal valine of Hb. The presence of other blood components, such as amino acids, may affect the formation of chemical–Hb adducts. Further research is expected to elucidate the remaining unknown factors that affect the formation of chemical–Hb adducts.

## 1. Introduction

In recent years, the presence of glycidol fatty acid esters (GEs) has been detected in foods such as cooking oil. When GEs are ingested as part of the diet, they are decomposed by the action of lipase to produce glycidol [1]. As glycidol is known as a mutagen and rodent carcinogen [2], the presence of GEs in foods has become a major societal problem. Given the concerns about the effects of mutations and carcinogens, such as glycidol, on human health, it is important to understand the level of dietary exposure to these chemical substances in humans and the early chemical effects in vivo. Therefore, screening methods using substance exposure indicators (biomarkers) have been developed. For a biomarker of a chemical substance, the target chemical substance itself or its metabolite present in blood or urine may be used. In particular, hemoglobin (Hb) adducts, produced when a chemical substance binds hemoglobin, the main component of erythrocytes, have attracted attention [3]. The formation of a hemoglobin adduct of glycidol occurs when glycidol binds to the N-terminal valine of the Hb structure [4,5]. *N*-(2,3-Dihydroxypropyl)valine is a hemoglobin adduct of glycidol and is used as a marker for internal exposure to glycidol [3,6,7,8].

Acrylamide is a mutagen/carcinogen formed via the Maillard reaction involving reducing sugars and/or carbonyl compounds and asparagine; it is found in high concentrations in foods such as potato chips, toast, and coffee. Acrylamide can react with DNA to induce mutagenicity and binds to the N-terminal valine of hemoglobin to form *N*-(*2*-carbamoylethyl)valine, which is a hemoglobin adduct of acrylamide [9,10,11,12,13].

In the present study, a modified Edman degradation method was used to quantify the glycidol– and acrylamide–Hb adducts. In this method, the N-terminal valine of the β chain of Hb to which a chemical substance is added is cleaved with the Edman reagent, fluorescein isothiocyanate (FITC), as a fluorescein thiohydantoin (FTH) derivative [14,15,16]. This method is called the “Adduct FI*R*E procedure™”, as the FITC reagent is used for the measurement of the adduct (*R*) produced from the electrophilic addition reaction by a modified Edman degradation method [14,15,16].

**Figure 1:**
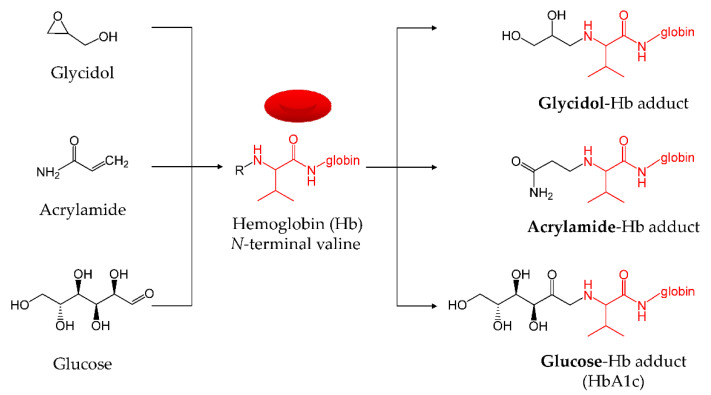
Chemicals that form adducts with the N-terminal valine of Hb.

Simultaneous exposure to substances that form an adduct with Hb, such as glycidol and acrylamide, may affect the formation of each individual hemoglobin adduct. Glucose also irreversibly binds to the N-terminal valine of hemoglobin to produce HbA1c (glycated Hb) [17]. It is possible that glucose and other chemicals, such as glycidol and acrylamide, compete for the N-terminal valine of Hb (Figure 1). The blood glucose level in people with diabetes is higher than that of healthy individuals; diabetes is diagnosed when the ratio of HbA1c in Hb is 6.5% or higher [18]. In patients with diabetes, changes in the concentration of blood components, such as glucose and protein, can impact the formation of hemoglobin adducts. Diabetic ketoacidosis (DKA) occurs when a patient with diabetes begins to exhaust their body’s supply of insulin. During DKA, the pH of blood is low, mainly because the bicarbonate buffer is exhausted; that is, the bicarbonate concentration drops [19]. It is expected that the amount of chemical substance–Hb adduct produced will fluctuate owing to changes in the conditions due to the onset of diabetes. However, there have been no reports on the formation of chemical substance–Hb adducts that actually consider the physiological conditions at the onset of diabetes.

In this study, the effects of glycidol concentration, pH, reaction time, coexistence of glucose, glycated Hb, serum albumin, and combined exposure of acrylamide on glycidol–Hb adduct formation were examined in vitro. Then, for the in vivo assay, glycidol and acrylamide were orally administered to ICR mice, and the amount of each Hb adduct in the blood was measured after 24 h. Next, in the ex vivo test, a mouse model of type I diabetes was established, the blood from the mice was exposed to glycidol and acrylamide, and the amount of each Hb adduct was compared. To investigate the difference between in vitro and in vivo results, we also examined amino acid residues other than the N-terminal valine of Hb and analyzed the interaction between Hb and glycidol or acrylamide using the protein thermal shift assay.

## 2. Materials and Methods

### 2.1. Chemicals

FITC (purity ≥ 90.0%), glycidol (purity 96.0%), human hemoglobin lyophilized powder (Product Number H7379), and human serum albumin lyophilized powder (purity ≥ 96.0%) were obtained from Sigma-Aldrich (St. Louis, MO, USA). Acrylamide (purity 95.0%) was obtained from FUJIFILM Wako Pure Chemical Industries Ltd. (Osaka, Japan). L-Valine-(^13^C_5_) (purity 98.0%), used for the synthesis of the glycidol internal standard *N*-(2,3-dihydroxypropyl)-(^13^C_5_)-valine, and L-*d*8-valine (purity 98.0%), used for the synthesis of the acrylamide internal standard fluorescein-5-[4-*d*7-isopropyl-3-(2-carbamoylethyl)-2-thioxo-imidazolidin5-one] (AA-*d*7-Val-FTH), was obtained from Cambridge Isotope Laboratories, Inc. (Tewksbury, MA, USA). All other chemicals and solvents used were of analytical grade.

### 2.2. Preparation for Glycidol–Hb Adduct Formation under Various Conditions (In Vitro)

#### 2.2.1. Effect of Glycidol Concentration on Glycidol–Hb Adduct Formation

Human Hb (final concentration 15 mg/mL) and glycidol (final concentrations 0, 5, 10, 50, 100, 200, 250, 300, 400, and 500 mM) were dissolved in 0.1-M phosphate buffer (pH 7.4). The solution (final volume 0.5 mL) was reacted at 37 °C for 18 h using a shaker (190 rpm).

#### 2.2.2. Effect of Reaction Time on Glycidol–Hb Adduct Formation

Human Hb (final concentration 15 mg/mL) and glycidol (final concentration 200 mM) were dissolved in 0.1-M phosphate buffer (pH 7.4). The solution (final volume 0.5 mL) was reacted at 37 °C for 0, 3, 6, 9, 12, 15, 18, 21, and 24 h using a shaker (190 rpm).

#### 2.2.3. Effect of Glucose on Glycidol–Hb Adduct Formation

Human Hb (final concentration 15 mg/mL); glycidol (final concentration 200 mM); and glucose (final concentrations 0, 1, 5, 20, and 100 mM) were dissolved in 0.1-M phosphate buffer (pH 7.4). The solution (final volume 0.5 mL) was reacted at 37 °C for 18 h using a shaker (190 rpm).

#### 2.2.4. Effect of Glycated Hb on Glycidol–Hb Adduct Formation

Human Hb (final concentration 15 mg/mL) and glucose (final concentrations 0, 5, 20, and 100 mM) were dissolved in 0.1-M phosphate buffer (pH 7.4). The solution (final volume 0.5 mL) was passed through a 0.22-µm sterile filter and then reacted at 37 °C for 7 days. After the reaction, the solution was placed in a cellulose tube (molecular weight cut-off 25,000) and dialyzed for 24 h. The dialyzed glycated Hb solution and glycidol (final concentration 200 mM) were then mixed (final volume 0.5 mL) and reacted at 37 °C for a further 18 h.

#### 2.2.5. Effect of pH on Glycidol–Hb Adduct Formation

Human Hb (final concentration 15 mg/mL) and glycidol (final concentration 60 mM) were dissolved in 0.1-M phosphate buffer at pH 6.6, 6.8, 7.0, 7.2, and 7.4. The solution was reacted at 37 °C for 18 h using a shaker (190 rpm).

#### 2.2.6. Effect of Serum Albumin on Glycidol–Hb Adduct Production

Human Hb (final concentration 15 mg/mL); glycidol (final concentration 60 mM); and serum albumin (final concentrations 0, 0.5, 1.0, 5.0, and 10.0 mg/mL) were dissolved in 0.1-M phosphate buffer (pH 7.4). The solution (final volume 0.5 mL) was reacted at 37 °C for 18 h using a shaker (190 rpm).

### 2.3. Preparation for Hb Adduct Formation in Combined Exposure to Glycidol and Acrylamide

#### 2.3.1. Effect of Acrylamide Concentration on Hb Adduct Formation following Combined Exposure (In Vitro)

Human Hb (final concentration 15 mg/mL); glycidol (final concentration 200 mM); and acrylamide (final concentrations 0, 100, 200, 500, and 1000 mM) were dissolved in 0.1-M phosphate buffer (pH 7.4). The solution (final volume 0.5 mL) was reacted at 37 °C for 18 h using a shaker (190 rpm).

#### 2.3.2. Effect of Glycidol Concentration on Hb Adduct Formation following Combined Exposure (In Vitro)

Human Hb (final concentration 15 mg/mL); acrylamide (final concentration 100 mM); and glycidol (final concentrations 0, 50, 100, 150, and 200 mM) were dissolved in 0.1-M phosphate buffer (pH 7.4). The solution (final volume 0.5 mL) was reacted at 37 °C for 18 h using a shaker (190 rpm).

#### 2.3.3. Hb Adduct Formation in Mice Exposed to Glycidol and Acrylamide (In Vivo)

Institute of Cancer Research (ICR) mice (Japan SLC, Hamamatsu, Japan, 5 weeks old, male, *n* = 5) were orally administered a solution of glycidol and acrylamide (2.0 mmol/kg b.w. each). After 24 h, whole blood was collected. The blood was centrifuged (1000× *g*, 5 min) to remove plasma, erythrocytes were added to physiological saline, and the mixture was centrifuged again (1000× *g*, 5 min). Prior to FITC derivatization, MilliQ water was added to the sample to induce hemolysis.

### 2.4. Hb Adduct Formation in Blood from Mice with Diabetes Mellitus (DM) Reacted with Glycidol and Acrylamide (Ex Vivo)

ICR mice (5 weeks old, male, *n* = 5) were intraperitoneally administered with an aqueous streptozotocin solution (200 mg/kg b.w.) to induce diabetes. After 2 weeks, fasting blood glucose was measured to confirm the increase in blood glucose level and that the mouse model of type I DM had been established. Glycidol or acrylamide (200 mM, pH 7.4) was added to blood (200 µL) taken from the model mice and reacted at 37 °C for 18 h.

### 2.5. Pretreatment for Hb Adduct Measurement

The solutions after each reaction of Section 2.2, Section 2.3 and Section 2.4 were heated at 37 °C for 18 h after adding FITC/*N*,*N*-dimethylformamide (5 mg/30 µL). Acetonitrile was added to the reaction mixture, which was centrifuged (10,000× *g*, 10 min), and then, a solution of 0.5-M ammonium hydroxide solution was added to the supernatant. This solution was added to the Oasis MAX cartridge (Waters, Milford, MA, USA), and the cartridge was washed with acetonitrile, water, and 0.5% cyanoacetic acid/water and then eluted with 0.25% cyanoacetic acid/acetonitrile. The eluate was concentrated and dried by nitrogen purging and then dissolved in 0.1% formic acid/acetonitrile:water (1:1) to prepare the final sample.

### 2.6. Determination of Hb Adduct by LC-MS/MS

The LC-MS/MS system comprised an HPLC Prominence system (Shimazdu, Kyoto, Japan) and an API2000 triple-quadrupole mass spectrometer (AB SCIEX, Tokyo, Japan). The HPLC MS analysis conditions and preparation of the glycidol internal standard *N*-(2,3-dihydroxypropyl)-(^13^C_5_) valine were as previously reported [20]. The acrylamide internal standard AA-*d*7-Val-FTH was made by the method of Von Stedingk et al. [14]. Chromatographic separation was achieved by a L-column ODS (150 mm × 2.1 mm, 5 µm; Chemicals Evaluation and Research Institute, Tokyo, Japan). Solvent A was 0.1% formic acid in acetonitrile:water (1:4), and solvent B was 0.1% formic acid in acetonitrile:water (4:1). A gradient was applied from 0% B to 20% B for 10 min and then stepped up to 100% B over 5 min before re-equilibrating the column with the initial mobile phase. The flow rate was 0.2 mL/min, and the injection volume was 5 µL. The MS conditions were polarity; positive ion mode; curtain gas, 20.0 psi; collision gas thickness, 6; temperature, 500 °C; gas supply 1, 60.0 psi; gas supply 2, 60.0 psi; and ion spray voltage, 5500 V. The detection limit (LOD) was set to a peak height three times the noise. Each target was evaluated by an analysis of calibration curve samples from five concentrations or three concentrations, including the internal standard (R’ = 0.999). Three analyses yielded an average coefficient of variation of 10% or less. The LC-MS/MS acquisition parameters (MRM mode) for the Hb adduct are shown in Table 1.

### 2.7. Interaction of Chemicals with Hemoglobin Using Thermal Shift Assay

#### 2.7.1. Interaction between Hb and Glycidol or Acrylamide

Human Hb (final concentration 0.125 mg/mL) and glycidol or acrylamide (final concentrations 0, 10, 100, 200, and 400 mM) were dissolved in 0.1-M phosphate buffer (pH 7.4). The solution (final volume 0.5 mL) was reacted at 37 °C for 3 h.

#### 2.7.2. Effect of Glucose on the Interaction of Hb with Glycidol or Acrylamide

Human Hb (final concentration 0.375 mg/mL) and glucose (final concentration 800 mM) were dissolved in 0.1-M phosphate buffer (pH 7.4). The solution (final volume 0.5 mL) was passed through a 0.22-µm sterile filter and then reacted at 37 °C for 2 days. The glycated Hb solution (75 μL) and a solution of 800-mM glycidol (25 μL) were mixed (final concentration of glycidol 200 mM) and further reacted at 37 °C for 3 h.

#### 2.7.3. Interaction with Hb during Combined Addition of Glycidol and Acrylamide

Human Hb (final concentration 0.125 mg/mL) and glycidol or acrylamide (final concentrations 0, 100, and 200 mM) were mixed (final volume 0.5 mL) and then incubated at 37 °C for 3 h.

#### 2.7.4. Protein Thermal Shift Assay

The thermal shift assay was performed using a Protein Thermal Shift™ Dye Kit (×1000; Thermo Fisher Scientific; Waltham, MA, USA). Each reacted sample (60 μL) was mixed with SYPRO Orange dye (×2) (60 µL), and 20 μL was dispensed into a tube. Using the StepOnePlus real-time PCR system (Applied Biosystems), the sample was thermally denatured by increasing the temperature from 25 °C to 99 °C at a rate of 0.16 °C/min, and the fluorescence intensity was measured. Data were analyzed using Protein Thermal Shift Software v1.0 (Applied Biosystems; Foster City, CA, USA) to determine the *T*_m_ value. The *T*_m_ value based on the Boltzmann fitting of the fluorescence/temperature raw data (*T*_m_B value) was used.

### 2.8. Statistical Analysis

The results were analyzed using one-way ANOVA, followed by the Tukey–Kramer test or Dunnett’s test using Microsoft Excel 2019 (Microsoft, Redmond, WA, USA). The significance level was set at *p* < 0.05, and all the experiments were replicated at least three times.

## 3. Results

### 3.1. Effect of Glycidol Concentration and Reaction Time on Glycidol–Hb Adduct Formation (In Vitro)

#### 3.1.1. Effect of Glycidol Concentration on Glycidol–Hb Adduct Formation

**Figure 2:**
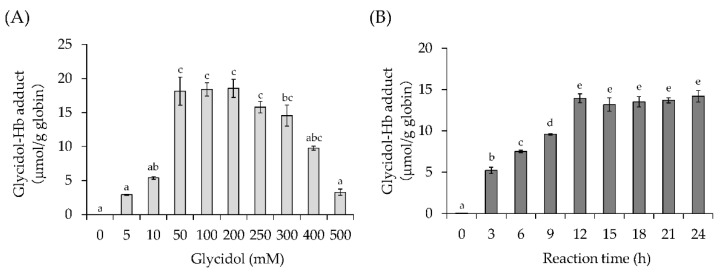
Effect of glycidol concentration and reaction time on glycidol–hemoglobin (Hb) adduct formation (in vitro). (**A**) Effect of glycidol concentration on glycidol–Hb adduct formation. Human Hb (final concentration 15 mg/mL) and different concentrations of glycidol were dissolved in 0.1-M phosphate buffer (pH 7.4). The solution was reacted at 37 °C for 18 h. (**B**) Effect of reaction time on glycidol–Hb adduct formation. Human Hb (final concentration 15 mg/mL) and glycidol (final concentration 200 mM) were dissolved in 0.1-M phosphate buffer (pH 7.4). The reaction solution was reacted at 37 °C for various times. Different letters indicate significant differences (Tukey–Kramer test, *p* < 0.05). The values represent the mean ± standard deviation (SD) of three independent experiments.

The effect of the glycidol concentration on the formation of the glycidol–Hb adduct was examined. Between 0 and 200-mM glycidol, the formation of the glycidol–Hb adduct increased in a concentration-dependent manner. The maximum concentration of the glycidol–Hb adduct formed was 18.5-μmol/g globin when 200 mM was added (Figure 2A). In contrast, when glycidol concentrations of 250 mM or more were added, a decrease in the amount of glycidol–Hb adduct formed was observed. Solutions containing 250 mM or more of glycidol were discolored, suggesting that Hb may be denatured (data not shown).

#### 3.1.2. Effect of Reaction Time on Glycidol–Hb Adduct Formation

The effect of reaction time on glycidol–Hb adduct formation was examined. Up to 12 h after the reaction of glycidol with Hb, there was a significant increase in the amount of glycidol–Hb adduct produced, but no significant change was observed after 12 h (Figure 2B). The maximum amount of glycidol–Hb adduct produced was 14.2-μmol/g globin for a reaction time of 24 h.

### 3.2. Effect of Factors on Glycidol–Hb Adduct Formation (In Vitro)

#### 3.2.1. Effect of Glucose on Glycidol–Hb Adduct Formation

**Figure 3:**
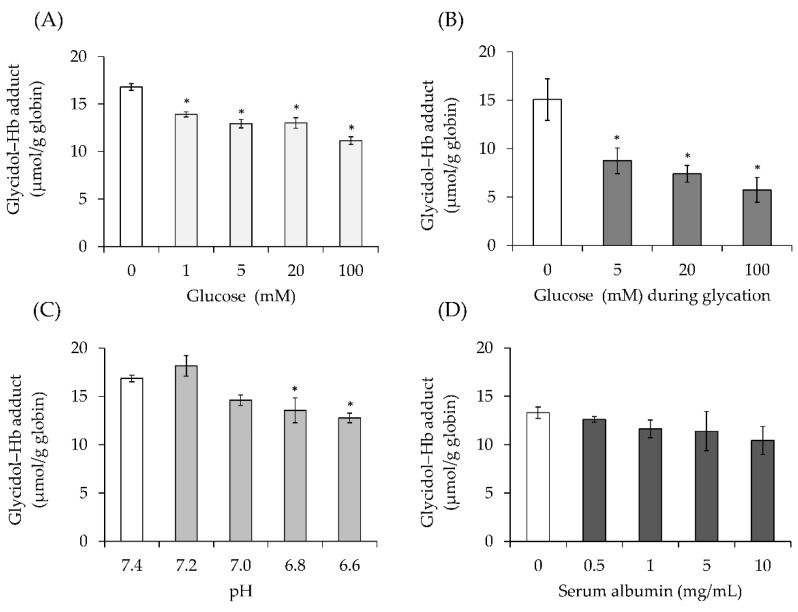
Effect of the factors on glycidol–hemoglobin (Hb) adduct formation (in vitro). Effect of (**A**) glucose, (**B**) glycated Hb, (**C**) pH, and (**D**) serum albumin on glycidol–Hb adduct formation. Each solution was dissolved in 0.1-M phosphate buffer (pH 7.4) and reacted at 37 °C for 18 h using a shaker. After FITC derivatization of the reaction solution, the glycidol–Hb adduct was measured by LC-MS/MS. The values represent the mean ± standard deviation (SD) of four independent experiments. * The significance level was *p* < 0.05 compared with glycidol (no additives, pH 7.4).

The effect of glucose on glycidol–Hb adduct formation was examined. The concentration of control glycidol–Hb adduct formed, without glucose added to Hb, was 16.8 μmol/g globin. In contrast, the addition of glucose to Hb at final concentrations of 1, 5, 20, and 100 mM significantly decreased the formation of glycidol–Hb adduct (13.9-, 12.9-, 13.0-, and 11.1-μmol/g globin, respectively) (Figure 3A).

#### 3.2.2. Effect of Glycated Hb on Glycidol–Hb Adduct Formation

The effect of glycated Hb on glycidol–Hb adduct formation was examined. The formation of the glycidol–Hb adduct was lower in the presence of Hb glycated with 5-mM glucose (8.8-μmol/g globin) as compared with the control Hb (15.1-μmol/g globin) (Figure 3B). Furthermore, the amount of glycidol–Hb adduct significantly decreased in the presence of the Hb glycated at 20 and 100 mM (7.4- and 5.8-μmol/g globin, respectively).

#### 3.2.3. Effect of pH on Glycidol–Hb Adduct Formation

The effect of pH on glycidol–Hb adduct formation was examined. The formation of the glycidol–Hb adduct was significantly decreased as the pH became more acidic (pH 7.4, 16.9-μmol/g globin; pH 7.2, 18.2-μmol/g globin; pH 7.0, 14.6-μmol/g globin; pH 6.8, 13.6-μmol/g globin; and pH 6.6, 12.8-μmol/g globin) (Figure 3C).

#### 3.2.4. Effect of Serum Albumin on Glycidol–Hb Adduct Formation

The effect of serum albumin on glycidol–Hb adduct production was examined. The control glycidol–Hb adduct formation, without serum albumin added to Hb, was 13.3-μmol/g globin. In contrast, the addition of serum albumin to Hb at final concentrations of 0.5, 1, 5, and 10 mg/mL decreased the formation of glycidol–Hb adduct (12.6-, 11.6-, 11.4-, and 10.4-μmol/g globin, respectively) (Figure 3D).

### 3.3. Effect of Combined Exposure to Chemicals on Hb Adduct Formation (In Vitro)

#### 3.3.1. Effect of Acrylamide Concentration on Hb Adduct Formation in Combination with Exposure to Glycidol

**Figure 4:**
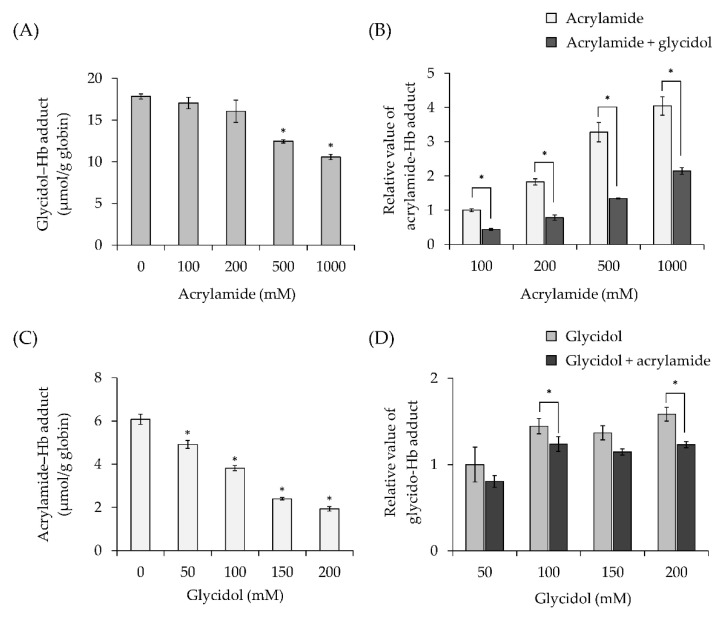
Effect of combined exposure to chemicals on Hb adduct formation (in vitro). (**A**) Effect of combined exposure to acrylamide on glycidol–Hb adduct formation. * The significance level was *p* < 0.05 compared with 0-mM acrylamide. (**B**) Relative value of acrylamide–Hb adduct formation with combined exposure of glycidol. * The significance level was *p* < 0.05 compared with the acrylamide alone at each concentration. (**C**) Effect of combined exposure to glycidol on acrylamide–Hb adduct formation. The significance level was *p* < 0.05 compared with 0-mM glycidol. (**D**) Relative value of glycidol–Hb adduct formation with combined exposure of acrylamide. * The significance level was *p* < 0.05 compared with glycidol alone at each concentration. The values represent the mean ± standard deviation (SD) of four independent experiments.

The effect of acrylamide concentration on Hb adduct formation in combination with exposure to glycidol was examined. Five concentrations of acrylamide (final concentrations 0, 100, 200, 500, and 1000 mM) were added to glycidol (200 mM), and the amounts of glycidol–Hb adduct and acrylamide–Hb adduct were measured. The formation of the glycidol–Hb adduct tended to decrease as the acrylamide concentration increased (Figure 4A). In combination with exposure to glycidol (200 mM), the formation of the acrylamide–Hb adduct tended to be lower compared with exposure to acrylamide alone (Figure 4B).

#### 3.3.2. Effect of Glycidol Concentration on Hb Adduct Formation in Combination with Exposure to Acrylamide

The effect of glycidol concentration on Hb adduct formation in combination with exposure to acrylamide was examined. Five concentrations of glycidol (final concentrations 0, 50, 100, 150, and 200 mM) were added to acrylamide (100 mM), and the formation of the glycidol–Hb adduct and the acrylamide–Hb adduct was measured. The formation of the acrylamide–Hb adduct tended to decrease as the glycidol concentration increased (Figure 4C). In combination with exposure to acrylamide (100 mM), the formation of the glycidol–Hb adduct tended to be lower compared with exposure to glycidol alone (Figure 4D).

### 3.4. Hemoglobin Adduct Formation in Mice Exposed to Glycidol and Acrylamide (In Vivo)

**Figure 5:**
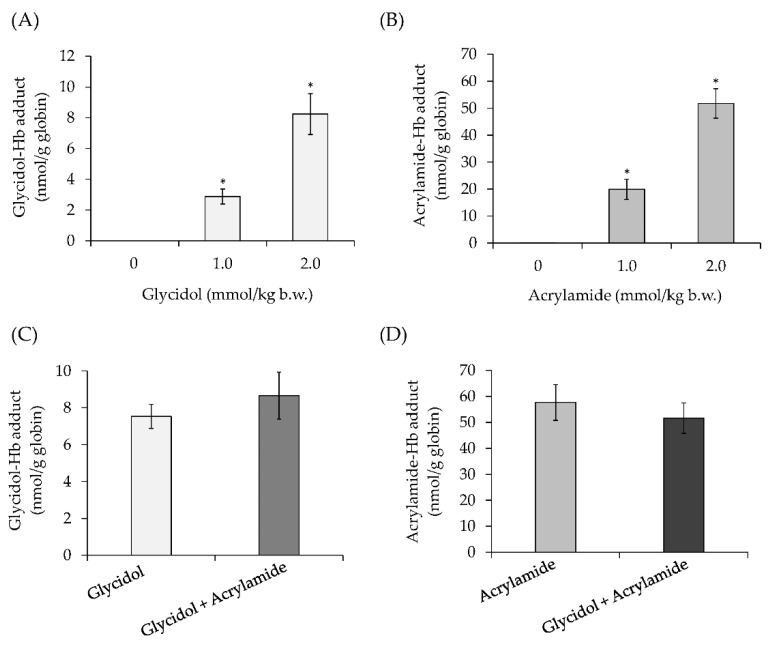
Hemoglobin (Hb) adduct formation in vivo in mice exposed to a combination of glycidol and acrylamide (in vivo). (**A**) Glycidol–Hb adduct formation following administration of glycidol alone to mice. (**B**) Acrylamide–Hb adduct formation following administration of acrylamide alone to mice. (**C**) Glycidol–Hb adduct formation in mice exposed to a combination of glycidol and acrylamide. (**D**) Acrylamide–Hb adduct formation in mice exposed to a combination of glycidol and acrylamide. * The significance level was *p* < 0.05 compared with the glycidol alone at each concentration. The values represent the mean ± standard deviation (SD) of four independent experiments.

The amount of glycidol or acrylamide–Hb adduct produced by the administration of glycidol or acrylamide alone to mice was examined. When glycidol or acrylamide was orally administered to mice, a concentration-dependent formation of glycidol– or acrylamide–Hb adducts was detected (Figure 5A,B). The formation of each Hb adduct following the combined administration of glycidol and acrylamide to mice was examined. There was no significant difference in the formation of glycidol–Hb adduct produced by glycidol alone (7.5-nmol/g globin) or glycidol + acrylamide (8.7-nmol/g globin) (Figure 5C). Similarly, there was no difference in the formation of acrylamide–Hb adduct produced by acrylamide alone (57.7-nmol/g globin) or glycidol + acrylamide (51.6-nmol/g globin) (Figure 5D).

### 3.5. Hb Adduct Formation during Exposure of Blood from Mice with Diabetes Mellitus to Glycidol or Acrylamide (Ex Vivo)

**Figure 6:**
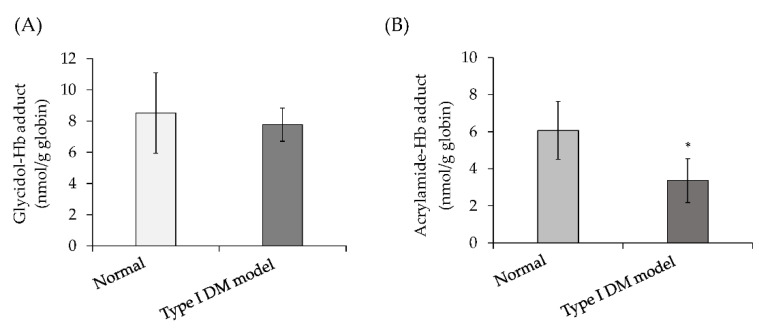
Hb adduct formation in blood from mice with diabetes mellitus (DM) reacted with glycidol or acrylamide (ex vivo). (**A**) Glycidol–Hb adduct formation in blood from the type I DM model mice. (**B**) Acrylamide–Hb adduct formation in blood from the type I DM model mice. * The significance level was *p* < 0.05 compared with the normal mouse blood. The values represent the mean ± standard deviation (SD) of four independent experiments.

The formation of the glycidol–Hb adduct was compared when glycidol was reacted with blood from normal mice and the model mice with type I DM (DM mice). The blood glucose levels were 96–101 mg/dL in normal mice and 248–486 mg/dL in the DM model mice. There was no difference in the formation of the glycidol–Hb adduct in the blood of normal mice (8.5-nmol/g globin) and the blood of DM mice (7.8-nmol/g globin) (Figure 6A). The formation of the acrylamide–Hb adduct was compared in the blood from normal mice and DM mice. A significant decrease in the formation of the acrylamide–Hb adduct was observed in the blood from DM mice (3.4-nmol/g globin) compared with the blood from normal mice (6.1-nmol/g globin) (Figure 6B).

### 3.6. Interaction of Chemicals with Hemoglobin Assessed by Thermal Shift Assay

#### 3.6.1. Interaction between Hb and Glycidol or Acrylamide

**Figure 7:**
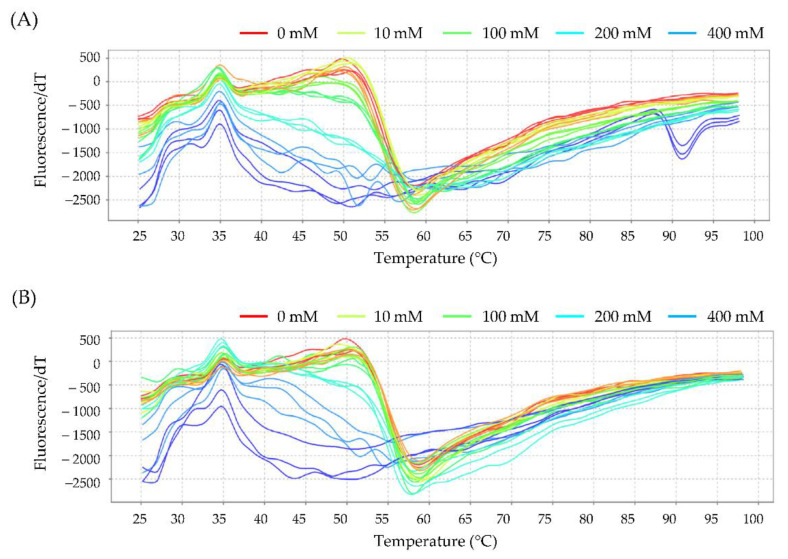
Melting profile of hemoglobin (Hb) with (**A**) glycidol or (**B**) acrylamide. The figures show the profile of the derivative of fluorescence emission as a function of temperature (dF/dT). Human Hb (0.125 mg/mL) and the ligand (0, 10, 100, 200, and 400 mM each for glycidol or acrylamide) were reacted at 37 °C for 3 h before analysis using the thermal shift assay.

The interaction of glycidol or acrylamide with Hb was analyzed using the protein thermal shift assay. In the thermal shift assay, when a fluorescent dye is added to a protein and heated, the dye binds to an exposed hydrophobic site, which causes it to fluoresce, and the change in fluorescence intensity can be measured to analyze the structural changes in the protein. The temperature (*T*_m_ value) at which the rate of increase in fluorescence intensity, obtained by differentiation over time, has the maximum value is the temperature at which the fluorescence intensity increases the most. At this temperature, the greatest changes in the protein structure occur. The melting profiles of Hb are shown in Figure 7, and the calculated *T*_m_ values are shown in Table 2. The *T*_m_ value for Hb alone was 60.03 ± 0.24 °C and decreased in a concentration-dependent manner as glycidol was added (Figure 7A). Similarly, *T*_m_ decreased as the concentration of acrylamide was increased (Figure 7B).

#### 3.6.2. Effect of Glucose on the Interaction of Hb with Glycidol or Acrylamide

**Figure 8:**
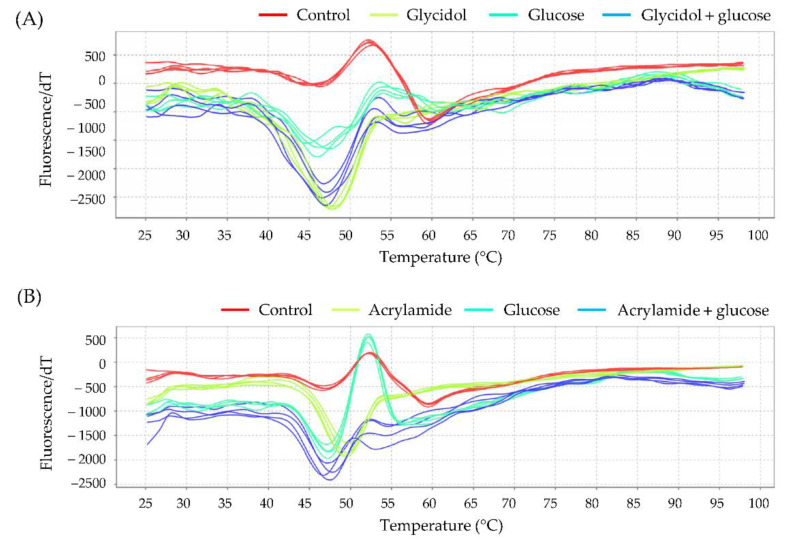
Melting profiles of hemoglobin (Hb) in the presence of glucose and (**A**) glycidol or (**B**) acrylamide. The figures show the profile of the derivative of fluorescence emission as a function of temperature (dF/dT). Glycidol or acrylamide was added after the preincubation of Hb and glucose.

The interaction of glycidol or acrylamide with Hb in the presence of glucose was analyzed using the protein thermal shift assay. The melting profiles of Hb are shown in Figure 8, and the calculated *T*_m_ values are presented in Table 3. The *T*_m_ value for Hb alone was 59.50 ± 0.11 °C. The *T*_m_ value of Hb to which glucose was added shifted to a lower temperature (57.70 ± 0.33 °C) compared with Hb alone, indicating that glucose interacted with Hb. The *T*_m_ value after the addition of glycidol or acrylamide to Hb preincubated with glucose (54.70 ± 0.09 °C and 54.85 ± 0.28 °C, respectively) was higher than the *T*_m_ value of Hb after the addition of glycidol or acrylamide alone (53.73 ± 0.22 °C and 53.18 ± 0.10°C, respectively). These results suggested that the reaction with glucose inhibited the interaction between Hb and chemicals.

#### 3.6.3. Interaction with Hb to the Combination of Glycidol and Acrylamide

**Figure 9:**
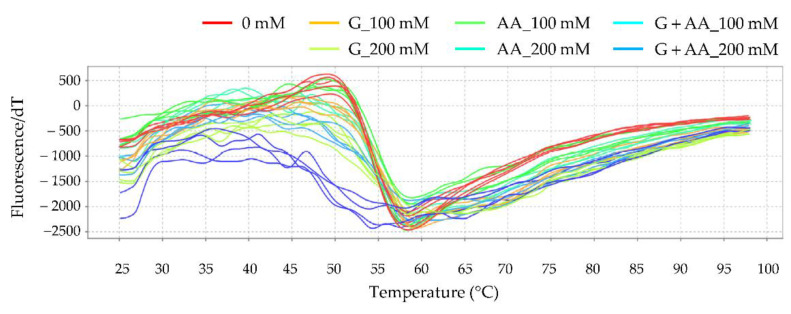
Melting profiles of hemoglobin (Hb) during combined exposure to glycidol and acrylamide. The figure shows the profile of the derivative of fluorescence emission as a function of temperature (dF/dT). Human Hb (0.125 mg/mL) and the ligand (0, 100, and 200 mM each for glycidol and acrylamide) were reacted at 37 °C for 3 h before analysis using the thermal shift assay.

The interaction of glycidol and acrylamide with Hb was analyzed using the protein thermal shift assay. The melting profiles of Hb are shown in Figure 9, and the calculated *T*_m_ values are presented in Table 4. The *T*_m_ value for Hb alone was 59.96 ± 0.10 °C. Although there was no significant difference in the *T*_m_ values when the concentration of each chemical substance was 100 mM, a significant difference was observed at 200 mM. Combined exposure to 200-mM glycidol and 200-mM acrylamide resulted in a significant difference in the *T*_m_ values. The combined exposure to glycidol and acrylamide caused stronger interactions with Hb.

## 4. Discussion

In this study, factors influencing the formation of Hb adducts of glycidol and acrylamide were analyzed using in vitro, ex vivo, and in vivo assays. The Hb concentration in the human blood is 140–180 mg/mL, but the reagent human Hb dissolved only up to about 20 mg/mL. In this study, the Hb concentration was set to 15 mg/mL in order to fully analyze the interactions with chemical substances. In addition, chemicals were used in an amount that produced the maximum amount of chemical-Hb adduct for the maximum amount of Hb to dissolve. When the effect of the glycidol concentration was examined in vitro, the formation of the glycidol–Hb adduct increased in a concentration-dependent manner up to 50 mM but did not an increase above that (Figure 2A). The saturation of valine adduct formation occurred only in the theoretical large molar excess of glycidol, suggesting that glycidol interacts with amino acids other than the N-terminal valine. At a glycidol concentration of 250 mM or higher, it was considered that the amount of glycidol–Hb adduct decreased owing to the change in the structure of Hb induced by the toxicity of the excessive concentration of glycidol.

When the effect of the reaction time between glycidol and Hb was examined, a significant increase was observed in the formation of the glycidol–Hb adduct for a reaction time of up to 12 h, but no significant change was observed after that (Figure 2B). A linear correlation between the glycidol and glycidol–Hb adduct levels was observed in rats at 24 h after the oral administration of glycidol, and glycidol was rapidly absorbed and bound to Hb in a dose-dependent manner [8]. In this study, it was also suggested that glycidol produces Hb adducts within 24 h in a dose-dependent manner in vitro. The second-order reaction rate constants *k_val_* for forming an adduct of Hb to the N-terminal valine were obtained from in vitro incubation with human hemoglobin. The *k_val_* for glycidol was determined according to Aasa et al. (2017) [21]. The level of glycidol adduct to Hb was plotted against the calculated in vitro dose, and *k_val_* was determined from the slope of the linear regression (data not shown). The *k_val_* for glycidol was determined up to 5.82-pmol/g Hb/μMh from the in vitro assay. The *k_val_* for glycidol in mouse, rat, and human blood was reported as 19.4- [21], 6.7-, and 5.6-pmol/g Hb/μMh [8]. Although there may be some effects depending on the test conditions and in vitro and in vivo differences, a *K_val_* value of the same order was obtained.

It is possible that components or conditions in the blood can affect the formation of Hb adducts of chemical substances. Therefore, the effect of glucose in the blood on the formation of the glycidol–Hb adduct was examined in vitro. The random plasma glucose was ≥200 mg/dL (≥11.1 mM), which is the definition of diabetes [22]. The formation of the glycidol–Hb adduct was significantly reduced by the addition of 1-, 5-, 20-, and 100-mM glucose, which is the same level of concentration as diabetics (Figure 3A). In addition, when glycidol was reacted with glycated Hb produced by the reaction of Hb and glucose, the formation of Hb adducts decreased depending on the glucose concentration (Figure 3B). Glucose is known to bind irreversibly to the N-terminal valine of Hb to produce glycated Hb, which is known as hemoglobin A1c, a biomarker of diabetes [17]. It was suggested that the competition between glycidol and glucose for the reaction with the N-terminal valine of Hb may reduce the formation of the glycidol–Hb adduct. Moreover, the formation of the glycidol–Hb adduct was decreased in acidic conditions (pH 7.2 or lower) (Figure 3C). It was reported that the pH of blood in streptozotocin-induced type 1 diabetic rats was 6.64 ± 0.30 [23]. At the onset of diabetes, DKA causes the blood to tend toward acidity [17], suggesting that the formation of the glycidol–Hb adduct may be reduced. The effect of serum albumin as a blood component on the formation of the glycidol–Hb adduct was examined. The formation of the glycidol–Hb adduct tended to decrease with the addition of serum albumin (Figure 3D). Serum albumin has been reported to bind to various chemicals [24,25] and is presumed to bind to glycidol. Depending on the disease state, the blood albumin concentration is decreased, or the pH of the blood is changed. These results suggest that the binding of glycidol to serum albumin may reduce the free glycidol and decrease the formation of the glycidol–Hb adduct.

The effect of combined chemical exposure on the formation of each Hb adduct was examined in vitro. When mixing two reactive chemicals (glycidol and acrylamide), they will compete for binding to the nucleophilic N-terminal valine of Hb. Therefore, glycidol and acrylamide were added to Hb, and the amount of each Hb adduct produced was measured in vitro. The results showed a significant decrease in the formation of each Hb adduct (Figure 4). When exposed to the combination of glycidol and acrylamide, the amount of the acrylamide–Hb adduct produced was larger than the decrease in the amount of the glycidol–Hb adduct produced (Figure 4B,D). These results suggest that glycidol may have a stronger ability to bind to Hb than acrylamide. The daily intakes of glycidol and acrylamide in humans were estimated to be 1.4 μg/kg b.w./day [26] and 0.02–1.53 μg/kg b.w./day [27], respectively. The daily intakes of both chemicals are considered to be similar. In addition, it was reported that the secondary rate constant of glycidol at the time of binding of Hb to the N-terminal valine was equivalent to that of acrylamide in vivo [8]. Therefore, the formation of Hb adducts in mice exposed to glycidol and acrylamide was examined in vivo.

When glycidol or acrylamide alone was administered to mice, the formation of each Hb adduct produced increased in a concentration-dependent manner (Figure 5A,B), consistent with previous reports [28,29]. There was no change in the amount of each Hb adduct produced when the combination of glycidol or acrylamide was administered to mice (Figure 5C,D), which was different from the in vitro results. The difference in the abundance of Hb and various components in the blood was considered as a factor that may influence the results of the in vitro assay. The normal range of hemoglobin is 13.5–18 g/dL (140–180 mg/mL) in adult males [30]. In contrast, the Hb concentration used in the in vitro assay of this study was reg/mL. In vitro, there are more chemical substances than Hb, and each chemical substance competes for the N-terminal valine of Hb, so it was considered that the amount of Hb adduct produced was changed. In vivo, more Hb was present than the chemical substance, and it did not compete with the N-terminal valine of Hb, suggesting that Hb adduct formation did not change. Furthermore, glycidol and acrylamide have different in vivo behaviors, such as metabolism, distribution, and excretion, which may affect the concentration of glycidol and the formation of their respective Hb adducts. However, in vivo in humans, unlike the mouse, it has been reported that there are other chemical substances (glyoxal, methylglyoxal, acrylic acid, 1-octen-3-one, etc.) that bind to the N-terminal valine of Hb [31]. Therefore, it is considered that glycidol and acrylamide may compete for binding to N-terminal valine-free Hb.

In the ex vivo assay, the formation of the glycidol– or acrylamide–Hb adduct was compared when glycidol or acrylamide was reacted with blood from normal mice and DM mice, respectively. There was no difference in the formation of the glycidol–Hb adduct in the blood of normal and DM mice (Figure 6A), but a significant decrease in the formation of the acrylamide–Hb adduct in the blood of DM mice was observed (Figure 6B). It was suggested that the physiological state in blood due to the onset of diabetes was more likely to affect the production of acrylamide–Hb adducts than glycidol–Hb adducts.

It was revealed that the Hb adduct formation with chemical substances fluctuated under the influence of various factors but that no change in Hb adduct formation was observed in vivo. It was suggested that the chemicals also interacted with amino acid residues other than the N-terminal valine of Hb. Therefore, the interaction of glycidol and acrylamide with residues other than the N-terminal valine of Hb was analyzed using the protein thermal shift assay. The effect of glucose on the interaction of glycidol or acrylamide with Hb was examined (Figure 7 and Table 1). When glycidol was added to Hb preincubated with glucose, the *T*_m_ value was less likely to shift to a lower temperature than when glycidol was added to Hb alone (Figure 8 and Table 3). These results suggested that the interaction between Hb and glycidol was inhibited in the presence of glucose. Furthermore, when the combination of glycidol and acrylamide was exposed to Hb, the *T*_m_ value was significantly shifted to a lower temperature (Figure 9 and Table 4). It was found that the combined exposure of the chemical substances resulted in stronger interactions with Hb. Therefore, glycidol and acrylamide may interact with binding sites other than the N-terminal valine of Hb.

The discrepancies observed between the in vitro, ex vivo, and in vivo results are considered to be caused by at least two factors. Hb in the blood is intracellular, while the in vitro studies were conducted using lyophilized purified protein. Since chemical substances need to enter the cells in order to react with hemoglobin in the blood, it is thought that the formation of Hb adducts was affected in the different test systems. In addition, concentrations of glycidol and acrylamide are much lower under physiological conditions than in vitro. The detoxification mechanism of the chemical substances and changes of the drug-metabolizing enzyme activity during disease may also be factors influencing the Hb adduct formation in vivo. Glycidol was reported to be metabolized to glycerol and to form a conjugation with reduced glutathione in vivo [32]. Acrylamide is not only metabolized to form a conjugation with reduced glutathione but is also oxidized by CYP2E1 to form the epoxide intermediate glycidamide [33]. In the future, it will be important to estimate the formation of Hb adducts of chemicals, considering the pharmacokinetics of the chemicals and the formation of bioactive species associated with DNA adducts inducing carcinogenesis.

## 5. Conclusions

The formation of glycidol– and acrylamide–Hb adducts was affected by various factors, and the amount formed changed in vitro. In contrast, the formation of the glycidol– and acrylamide–Hb adducts was unchanged in mice exposed to glycidol and acrylamide in vivo. In addition, glycidol and acrylamide also interacted with amino acid residues other than the N-terminal valine of Hb. It was suggested that the binding of the chemical substances to Hb changed the higher-order structure, altering the binding property of Hb to the N-terminal valine. However, the presence of other blood components, such as albumin and amino acids, as well as the status of any diseases, such as diabetes, may affect the formation of adducts between chemicals and Hb. Further research is expected to elucidate the various unknown factors that affect the formation of chemical–Hb adducts.

## Figures and Tables

**Table 1:** LC-MS/MS acquisition parameters in multiple reaction monitoring (MRM) mode for the Hb adduct.

Hb Adduct	Polarity [ESI]	Retention Time (min)	Precursor Ion (*m/z*)	Product Ion (*m/z*)	Regression Equation (*r*^2^)	LOD (pmol/g globin)	LOQ (pmol/g globin)	Recovery Rate (%)
G-Val-FTH *^1^	[M + H]^+^	9.45	563	390	*y* = 323,411*x* + 6187.2 (0.9998)	67.1	201.2	87
AA-Val-FTH *^2^	[M + H]^+^	9.70	560	390	*y* = 739,049*x* + 29,196 (0.9994)	57.7	173.1	92

*^1^ Glycidol–valine adduct FITC derivative. *^2^ Acrylamide–valine adduct FITC derivative.

**Table 2:** Melting temperature of hemoglobin exposed to glycidol or acrylamide.

	*T*_m_ (°C)				
	0 mM	10 mM	100 mM	200 mM	400 mM
Control	60.03 ± 0.24	−	−	−	−
Glycidol		60.50 ± 0.08	59.19 ± 0.19 *	57.53 ± 0.09 *	56.36 ± 0.10 *
Acrylamide		59.91 ± 0.17	59.48 ± 0.22 *	58.73 ± 0.07 *	56.49 ± 0.05 *

* *p* < 0.05 compared to the control.

**Table 3:** Melting temperature of hemoglobin in the presence of glucose and glycidol or acrylamide.

	*T*_m_ (°C)	
	Without Glucose	With Glucose
Control	59.50 ± 0.11 ^a^	57.70 ± 0.33 ^b^
Glycidol	53.73 ± 0.22 ^c^	54.70 ± 0.09 ^d^
Acrylamide	53.18 ± 0.10 ^c^	54.85 ± 0.28 ^d^

Different letters indicate significant differences (Tukey–Kramer test, *p* < 0.05).

**Table 4:** Melting temperature of hemoglobin with glycidol and acrylamide.

	*T*_m_ (°C)		
	0 mM	100 mM	200 mM
Control	59.96 ± 0.10	−	−
Glycidol		59.68 ± 0.20	58.67 ± 0.20
Acrylamide		60.09 ± 0.37	59.42 ± 0.39
Glycidol + acrylamide		59.96 ± 0.29	56.58 ± 0.08 *

* *p* < 0.05 compared to the control.

## Data Availability

The data presented in this work are available in the article.
